# Hemocholecyst Secondary to Gallbladder Polyp Rupture: A Report of a Rare Case

**DOI:** 10.7759/cureus.111507

**Published:** 2026-06-25

**Authors:** Alaa Kansoun, Nicolas Turrin

**Affiliations:** 1 General Surgery, Hôpital Saint Joseph, Marseille, FRA; 2 Surgery, Hôpital Saint Joseph, Marseille, FRA

**Keywords:** bleeding, cholecystectomy, gallbladder, hemocholecyst, polyp

## Abstract

Hemocholecyst is a rare condition that is frequently misdiagnosed due to its atypical clinical presentation. In fact, with the widespread use of ultrasonography, gallbladder polyps are increasingly well documented. However, hemocholecyst is rarely reported in association with gallbladder polyps, particularly in the absence of anticoagulant therapy, gallstones, malignancy, or prior radiologic interventions. We report a case of a gallbladder polyp complicated by rupture leading to hemocholecyst. Hence, hemocholecyst may occur, particularly when a polyp is pedunculated and exhibits marked vascularity.

## Introduction

The term hemocholecyst (HC) was first introduced by Fiessinger and colleagues in 1938 [1]. It is defined as the presence of blood within the gallbladder lumen [2]. The most common causes of HC are gallstones and malignancy [3]. However, traumatic events and interventional procedures may also result in HC [4]. In addition, HC may occur secondary to hemobilia, which is defined as bleeding within the biliary tree [5,6]. Anticoagulant therapy is another major contributing factor [2]. In contrast, HC secondary to gallbladder polyps has been rarely reported. We therefore present this rare case of HC in a patient under follow-up for a gallbladder polyp. This report was prepared in accordance with the Declaration of Helsinki and local policy on case reports. The consent also covers publication of images.

## Case presentation

A 63-year-old man presented to our clinic with abdominal pain. His medical history included arterial hypertension, hypercholesterolemia, and depression. He had previously undergone Nissen fundoplication for hiatal hernia 10 years earlier, which was complicated by dysphagia and subsequently treated with endoscopic esophageal dilation. He was also an active tobacco smoker.

The patient had been under ultrasound surveillance for a gallbladder polyp measuring 11 mm in September 2024 that showed progressive increase in size; therefore, an elective cholecystectomy had been scheduled in this context. The polyp attained 16.9 x 12.4 x 9.5 mm in diameter with a significant vascularized pedicle in November 2025 (Figure 1).

**Figure 1:**
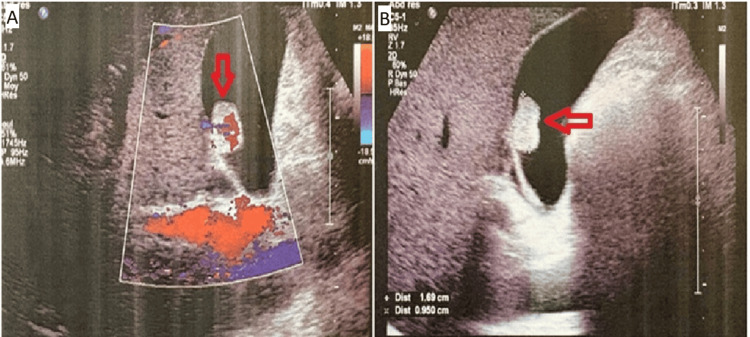
(A, B) Ultrasound image showing a well-vascularized gallbladder polyp identified by Doppler phase showing red and blue colors through the polyp. The arrow indicates the polyp.

On physical examination, the patient was afebrile with stable vital signs. There was no Murphy’s sign, and abdominal palpation revealed a soft, non-tender abdomen. Notably, the abdominal pain resolved after administration of simple analgesics (paracetamol) and antispasmodic agents (trimethylphloroglucinol/phloroglucinol).

Given the suspicion of biliary colic, laboratory investigations, including liver function tests (LFTs), were performed to rule out acute cholecystitis. Blood test results are summarized in Table 1.

**Table 1:** Summary of the laboratory results during hospitalization.

Biology	Result	Unit	Reference range
Hemoglobin	14	g/dL	13-18
C-reactive protein	3.1	mg/L	<5
White blood cells	8	×10⁹/L	4-11
Aspartate aminotransferase (AST)	364	U/L	10-50
Alanine aminotransferase (ALT)	182	U/L	10-50
Gamma-glutamyl transferase	342	U/L	<60
Alkaline phosphatase	Normal	U/L	40-129
Bilirubin	Normal	µmol/L	<24
Creatinine	Normal	µmol/L	59-104

A computed tomography (CT) scan demonstrated gallbladder distension with dense intraluminal contents consistent with biliary sludge (Figure 2).

**Figure 2:**
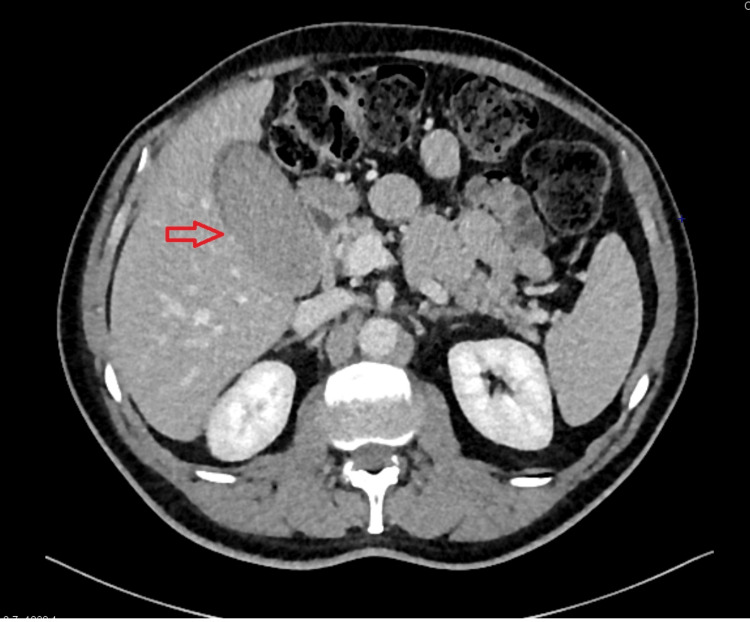
Computed tomography image demonstrating a distended gallbladder without signs of infiltration, infection, or biliary tract dilatation. The arrow indicates the gallbladder.

Further evaluation with ultrasonography was therefore recommended. Abdominal ultrasound revealed heterogeneous gallbladder contents without significant wall thickening, findings suggestive of HC, with no involvement of the common bile duct (CBD) (Figure 3). Table 2 is a dated timeline showing the corresponding presentation and workup.

**Figure 3:**
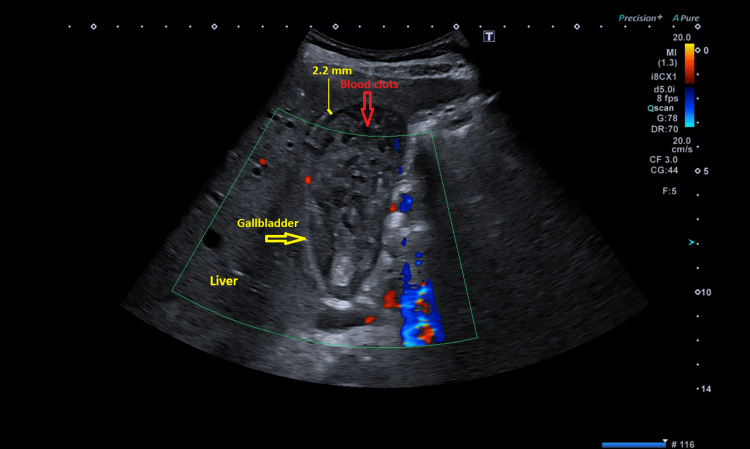
Ultrasound image showing heterogeneous gallbladder contents without significant wall thickening. The arrow indicates the heterogeneous content inside the gallbladder. In addition, the gallbladder wall was within normal ranges: 2.2 mm.

**Table 2:** Dated timeline showing the corresponding presentation and workup relative to day 0, which corresponds to the operation day. 0: day of the operation; -: time before the operation; SGOT: serum glutamic oxaloacetic transaminase; SGPT: serum glutamic pyruvic transaminase; GGT: gamma-glutamyl transferase; CRP: C-reactive protein; WBC: white blood cell; CT: computed tomography; CBD: common bile duct

	Information	Date
Presentation	Incidental finding	Year -2
Ultrasound	11-12 mm gallbladder polyp	Year -2
Follow-up ultrasound 2	Increase in size to 16.9 x 12.4 x 9.5 mm in diameter documenting a vascularized pedicle	Year -1.5
Laboratory results	SGOT 40 IU/L, SGPT 36 IU/L, GGT 50 IU/L, alkaline phosphatase 80 IU/L, total bilirubin 12 µmol/L, CRP 3 mg/L, WBC 5 x 10^9^/L	Year -1
Presentation 2	Abdominal pain	Day -1
Ultrasound 3	Heterogeneous content in the gallbladder suggesting hemobilia	Day -1
Abdomino-pelvic CT scanner	Dense gallbladder suggesting acute cholecystitis	Day -1
Laboratory results 2	Hemoglobin 14 g/dL, SGOT 364 IU/L, SGPT 182 IU/L, GGT 342 IU/L, alkaline phosphatase 97 IU/L, total bilirubin 12.2 µmol/L, CRP 1 mg/L, WBC 8 x 10^9^/L	Day -1
Operation	Cholecystectomy with intraoperative cholangiography	Day 0
Cholangiography	Intraoperative: vacuity of CBD	Day 0
Laboratory results 3	Hemoglobin 14.1 g/dL, SGOT 63 IU/L, SGPT 123 IU/L, GGT 281 IU/L, alkaline phosphatase 122 IU/L, total bilirubin 12 µmol/L, CRP 13 mg/L, WBC 6 x 10^9^/L	
Pathology report	Chronic cholecystitis. Absence of polyp	Day 14
Pathology relecture	No polyp or traces identified	Day 18
Postoperative follow-up	Asymptomatic, hemoglobin 14.1 g/dL, SGOT 50 IU/L, SGPT 43 IU/L, GGT 55 IU/L, alkaline phosphatase 98 IU/L, total bilirubin 10 µmol/L, CRP 1 mg/L, WBC 6 x 10^9^/L	Day 30

Surgical management was chosen, and the patient underwent a laparoscopic cholecystectomy with intraoperative cholangiography (IOC). It was a standard laparoscopic cholecystectomy using four trocars and following the critical view of safety rule. Precisely, no active bleeding, gallbladder wall abnormalities, or bile duct blood were identified at IOC. In addition, the cholangiography showed that the biliary tree was intact, with no evidence of obstruction (Figure 4). The gallbladder specimen was opened ex vivo for macroscopic examination. Not to mention that no significant intra-op findings were identified, such as wall inflammation, necrosis, mucosal ulceration, or the presence of active oozing. As shown in Figure 5, the gallbladder was filled with blood clots. After complete removal of the clots, no polyp or other macroscopic abnormalities were identified, and the entire specimen was sent to histological analysis.

**Figure 4:**
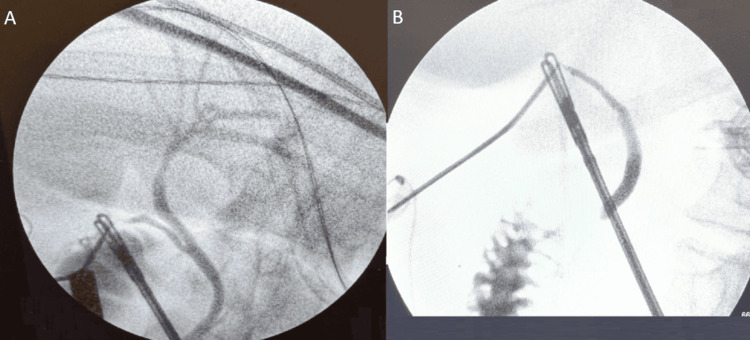
(A, B) Intraoperative cholangiography showing an intact common bile duct confirming vacuity with no obstruction.

**Figure 5:**
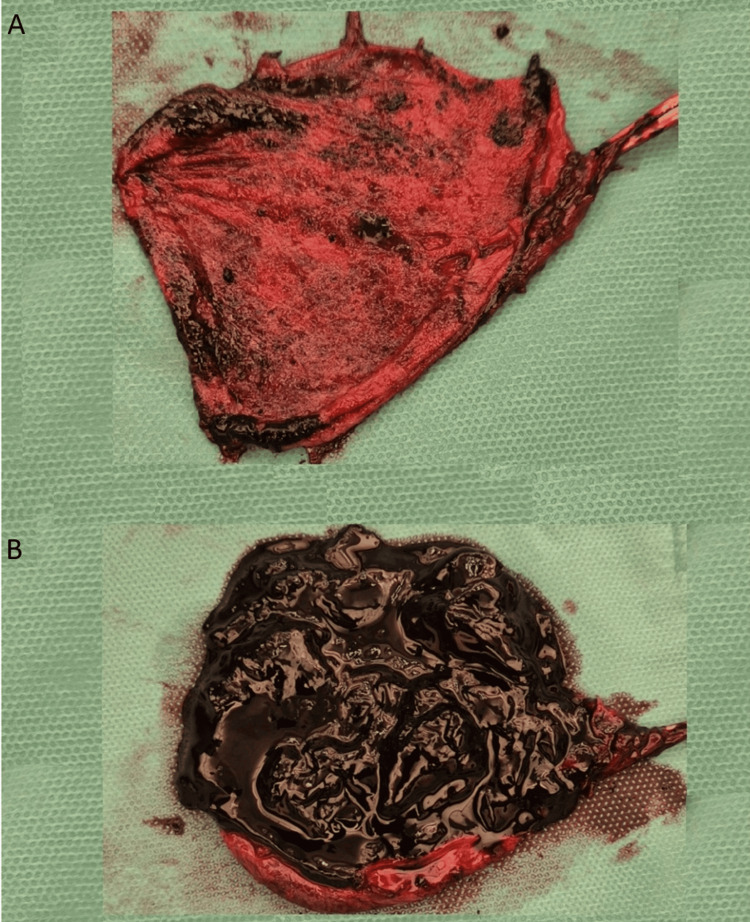
(A, B) Gallbladder specimen opened ex vivo, showing blood clots and absence of a polyp.

The postoperative period was unremarkable, and the patient was discharged on day 2. Concerning the histology, the examination confirmed chronic cholecystitis without evidence of malignancy or gallbladder polyps. The pathologist was contacted for re-evaluation, and repeat examination of the specimen again revealed no polyp. Precisely, the microscopic examination revealed a mucosa lined with preserved villi, which were covered by columnar cells. These cells were regular, without atypia. The lamina propria was fibrocollagenous and contained congested capillaries. The lymphoplasmacytic inflammation was mild. Moreover, the muscularis propria was moderately thickened, and the subserosa contained hyperplastic nerve endings and congested arteriolar vessels. In addition, no erosive areas were observed. The repetitive exhaustive testing did not find any polyp; hence, we raised the hypothesis of a ruptured polyp with auto-detachment as a mechanism explaining our case.

At the one-month postoperative follow-up, the patient was asymptomatic, and LFTs had returned to normal. The patient was reassured and satisfied with the management provided and the explanation clarifying the diagnosis.

## Discussion

Although the treatment of HC is relatively well established, its diagnosis and underlying etiology remain challenging. Recent publications suggest that HC itself may represent an indication for cholecystectomy [7]. Regarding etiology, HC has been reported in association with hemoperitoneum secondary to antiplatelet and anticoagulant therapy [2]. In addition, interventional procedures such as radiofrequency ablation for liver tumors have been identified as potential causes of HC [4]. Several studies have also described an association between HC and gallbladder malignancy [2,8,9].

Unlike our case, clinical presentations including melena and jaundice have been reported in association with HC due to hemobilia [5,6,10]. None of these studies established a correlation between gallbladder polyps and the risk of HC. However, a case reported in India in 2010 described hemobilia and HC secondary to a gallbladder adenoma [5]. Another case reported in 2013 described a rare presentation of hemobilia in which the gallbladder polyp was considered the only plausible cause [6]. Table 3 provides a brief comparison between the reported cases of gallbladder polyps resulting in bleeding.

**Table 3:** Brief table comparing the polyp characteristics and clinical outcomes of the reported cases of gallbladder polyps.

	Polyp characteristics	Clinical outcomes
Wani et al. [5]	NA	Acute cholecystitis
Juul Nielsen et al. [6]	2 cm	Hemobilia
Kansoun and Turrin (current study)	1.6 cm	Biliary colics

The patient was known and followed for his gallbladder polyp; thus, it was unlikely that an adherent clot with motion artefact or an intramucosal vascular lesion was responsible for his diagnosis. Moreover, the patient did not suffer from vascular lesions or cirrhosis responsible for gallbladder varices. Moreover, the patient did not take any anticoagulants, antiplatelet agents, or herbal medications associated with bleeding risk. On the other hand, no inflammatory syndrome was identified during his hospitalization, which ruled out acute cholecystitis or, in this context, acute hemorrhagic cholecystitis. Moreover, the patient did not present any sign of jaundice or gastrointestinal bleeding, which made a diagnosis of hemobilia doubtful. We reinforced our hypothesis of HC by the normal IOC. The perturbation of LFT was most probably due to the migration of a blood clot into the CBD.

Given this potential risk, we reviewed the most recent guidelines for the management of gallbladder polyps. Specifically, we examined the updated recommendations derived from the 2017 guidelines developed jointly by the European Society of Gastrointestinal and Abdominal Radiology (ESGAR), the European Association for Endoscopic Surgery and Other Interventional Techniques (EAES), the International Society of Digestive Surgery-European Federation (EFISDS), and the European Society of Gastrointestinal Endoscopy (ESGE) [11]. According to these guidelines, cholecystectomy is indicated for gallbladder polyps measuring ≥10 mm in patients fit for surgery, for polyps that increase in size by ≥2 mm during follow-up, and for symptomatic patients. Additional indications include polyps measuring 6-9 mm in patients with risk factors such as age > 60 years, primary sclerosing cholangitis, Asian ethnicity, sessile morphology, or focal gallbladder wall thickening > 4 mm [12].

Based on the analysis of our case, we hypothesize that rupture of the polyp stalk resulted in intraluminal hemorrhage and subsequent necrosis of the polyp or migration through the CBD and then the duodenum, explaining its absence on both macroscopic and histopathological examination. In light of this case, the risk of hemorrhage associated with gallbladder polyps should be considered. Particular attention should be paid to well-vascularized polyps. We are convinced that well-vascularized polyps may rarely be complicated by hemorrhage, warranting close surveillance and individualized surgical decision-making in selected patients.

Study limitations

This is a single-case report that highlights the potential risk of hemorrhage associated with gallbladder polyps. Without histological evidence, the hypothesis remains debatable. Larger studies are needed to confirm this observation and to better define optimal management strategies.

## Conclusions

Rupture of a gallbladder polyp may occur, particularly when the polyp’s pedicle is markedly vascularized, leading to HC. Precisely, the mechanism of bleeding can be due to vascular erosion, torsion, or ulceration. We suggest that vascularity, rather than size alone, may be a critical factor in predicting complications. Reporting this rare complication is essential to improve understanding of this entity and to emphasize the importance of appropriate management, although alternative etiologies must be carefully excluded before establishing causation of HC.
